# Desmin detection by facile prepared carbon quantum dots for early screening of colorectal cancer

**DOI:** 10.1097/MD.0000000000005521

**Published:** 2017-02-03

**Authors:** Chang-feng Li, Zhen-kun Yan, Li-bo Chen, Jing-peng Jin, Dan-dan Li

**Affiliations:** a Department of Endoscopy Center; b Department of Ultrasonography, China-Japan Union Hospital of Jilin University, Changchun, Jilin, P.R. China.

**Keywords:** carbon quantum dots, colorectal cancer, desmin, early screening, photoluminescence

## Abstract

Th aim of this study was to develop a new facile chemical method for early screening of colorectal cancer.

The -C(O)OH groups modified Carbon Quantum Dots (CQDs) were prepared by an facile innovative route of acid attacking on carbon nanotubes (CNTs). The -C(O)OH groups were further transported into -C(O)Cl groups by SOCl_2_ treating. The obtained ClCQDs were conjugated onto the anti-Desmin, which were applied for testing the Desmin concentration in serum by using linearly fitted relationship with photoluminescence (PL) intensity.

The obtained carbon quantum dots are quasispherical graphite nanocrystals with photoluminescence at about 455 nm. The Desmin with concentration of 1 ng/mL can lead to a decrease of PL intensity for anti-Desmin conjugated CQDs with good linearity. This assay had good specificity for Desmin with in interferential substances of immunoglobulin G (IgG), alpha fetoprotein (AFP), and carcinoembryoic antigen (CEA).

A new facile acid attack method was developed to prepare ClCQDs, which could conjugate onto the anti-Desmin for detection of Desmin in serum with high sensitivity and specificity. As the detection limit is lower than 1 ng/ mL, this work provides a promising strategy for the evaluation of colorectal cancer risk with low cost and excellent sensing performance.

## 1 Introduction

As one of the most common cancers, colorectal cancer (CRC) accounts for about 8% death of cancer all over the world. According to International Agency for Research on Cancer (IARC), CRC becomes the second leading cause in female's malignant tumors and the third in male's.^[1,2]^ Similar to other common malignant tumors, early detection, early diagnosis, and early accepted treatment could obviously enhance the outcome.^[3]^ It is generally acknowledged that the early detection and screening of tumor marker (TM) are critical for the cancer conquering. Serological detection of specific TM is widely applied for early diagnosis of CRC, owing to its good repeatability, high compliance, and small trauma. Moreover, the clinical value of serological testing depends on the validity of TM and the sensitivity of the detection technique. Carcinoembryonic antigen (CEA) is traditionally considered to be one of the most important for CRC evaluation.^[4,5]^ In almost all of the lesions of CRC patients, the expression of CEA would be increased, whose level had a positive correlation with the prognosis. However, the specificity and the sensitivity of CEA to CRC patients were estimated to be only 65% and 30%, respectively, and the early sensitivity is <20%.^[6]^ Therefore, CEA is not an ideal TM for CRC screening.

Desmin, an intermediate filaments protein type III, is one of the earliest expressed muscle-specific proteins, which could be detected in early phase of skeletal and cardiac muscle differentiation together with vimentin and nestin. Desmin-null mutant mice show disorganization of myofibril architecture in mechanically stressed striated muscles, including heart. Furthermore, Desmin gene mutations would lead to certain forms of muscular dystrophies, with or without cardiomyopathies.^[7]^ Studies have shown that the expression of Desmin is significantly increased in CRC and adenomas comparing with normal colonic tissues, indicating a potential new colon and rectal cancer serum marker.^[8]^ A high expression of Desmin in colon cancer patients is correlated with a decreased survival and poor prognosis, as it disrupts the tumor suppressive function of p53. Therefore, Desmin is also recognized as a novel prognostic predictor and therapeutic target for CRC. However, as enzyme-linked immunosorbent assays (ELISAs) and electrochemiluminescence (ELC) assays are sample-consuming and high-cost methods, it is very necessary to develop a simple and fast method with high sensitivity and specificity for the detection of Desmin in serum. The quantum dots probe may have much more advantages of fast, cost-effective, as well as facile operation, among others. The semiconductive quantum dots, such as CdTe, CdSe, among others, have been already applied for the in vitro diagnosis.^[9–11]^

Following the buckminsterfullerene (C60), carbon nanotubes (CNTs), and graphene, a new member in the carbon nanomaterial family, carbon quantum dots (CQDs) have recently attracted worldwide attention because of their great potential applications in various fields. Comparing with the traditional semiconductive quantum dots, an obvious advantage of these CQDs for biosensors is that they are much low toxicity, which is also potential for the labeling in vivo.^[12]^

CQDs offer unique advantages over traditional organic fluorescent agents and semiconducting quantum dots: they are cheaper, chemically inert, easy to functionalize, little toxic, and biocompatible.^[13]^ They also display stable and tunable photoluminescence, and they can function as electron donors and acceptors.^[14–17]^ A number of techniques afford CQDs, including laser ablation,^[18]^ pyrolysis,^[19]^ electrochemical synthesis,^[20]^ supported synthesis,^[21]^ acid oxidation,^[15]^ microwave-assisted synthesis,^[22]^ and hydrothermal synthesis.^[23]^ The synthetic approaches are classified as either “top-down” or “bottom-up” methods, depending on the precursors. The bottom-up methods involve the synthesis of CQDs from molecular precursors such as citric acid, glucose, and organic resins. However, the CQDs prepared by this strategy were usually very weak photoluminescence. Top-down methods refer to the preparation of CQDs from larger carbon materials, such as carbon fibers, graphite, and CNTs. Recently, researchers have employed oxidization of single-walled carbon nanotubes, multiwalled carbon nanotubes (MWCNTs), and graphite by mixed acids to obtain CQDs.^[12,13]^

In this article, the -C(O)OH groups modified CQDs were obtained from MWCNTs oxidation by acid attack. Then the resulting product was treated with SOCl_2_ to generate -C(O)Cl groups. Finally, anti-Desmin was covalently conjugated with the prepared CQDs to develop a novel assay for the detection of Desmin in the peripheral blood, and the concentration of Desmin in peripheral blood can be easily calculated by monitoring of fluorescence intensity quenching with high sensitivity.

## 2 Experimental

### 2.1 Reagents

MWCNTs were purchased from Beijing Nachen S&T Ltd., China. The active human Desmin full-length protein, anti-Desmin antibody, as well as immunoglobulin G (IgG), carcinoembryoic antigen (CEA), and alpha fetoprotein (AFP) were purchased from Abcam PLC (Cambridge, UK). Human peripheral samples were provided by China-Japan Union Hospital of Jilin University, Changchun, Jilin, P.R. China. Other reagents purchased from Sinopharm Chemical Reagent Co, Ltd, China, were analytical grade and directly used without purification. The ultrapure water (18.2 MΩ cm) acquired from a Millipore Milli-Q system was used for preparing solutions.

### 2.2 Synthesis of CQDs

A total of 4 g of MWCNTs was added into a mixture of concentrated H_2_SO_4_ (300 mL) and HNO_3_ (100 mL). The resulting solution was sonicated for 2 hours, which was followed by reflux at 80°C ± 3°C under constant stirring for 8 hours. A light brown gas evolved. After cooling and centrifugation, the pH was adjusted to 8 with NaOH. The product was washed 4 or 5 times with distilled water, dried in vacuum at 70°C, and identified as carboxylated MWCNTs. Reflux in excess SOCl_2_ (25 mL for 0.1 g) and dimethylformamide (1 mL) at 70°C to 72°C for 24 hours converted the carboxyl groups of the carboxylated MWCNTs to carbonyl chloride groups. The product was separated by distillation, dried in a vacuum oven at 70°C, and identified as CQDs with carbonyl chloride. The CQDs functionalized with carbonyl chloride were filtered for purification and centrifuged at 3600 rpm for 1 hour and the products were named as ClCQDs.

The morphology and composition of the products were investigated on a high-resolution transmission electron microscopy (HRTEM, JEOL JEM-2010). The LS55 spectrophotometer (Perkin-Elmer) was applied for the characterization of the photoluminescence (PL) properties.

### 2.3 Conjugation of anti-desmin to CQDs

The prepared ClCQDs were dissolved into 10 μL *N*-(3-dimethylaminopropyl)-*N*-ethylcarbodiimide hydrochloride (EDC), forming a 0.4 mol/L solution. Then, 10 μL 0.1 mol/L *N*-hydroxysuccinimide (NHS) was added with stirring. About 30 minutes later, 50 μL of anti-Desmin antibody with concentration of 10 μmol/L was added to the mixture and incubated for 60 minutes. A centrifugal ultrafiltration device was applied for purification of anti-Desmin conjugated CQDs and removing the excessive antibody.

The purified anti-Desmin conjugated CQDs were firstly redispersed into EDC solution at room temperature, and then diluted by 0.01 mol/L phosphate buffer solution (PBS, pH 7.2). The resulted solution had the final anti-Desmin conjugated CQDs concentration of 50 ng/mL, and was named as Solution C, whose PL intensities were recorded at λ_ex/em_ = 300/450 nm.

### 2.4 Desmin detection by anti-desmin conjugated CQDs

The Ethics Committee of China-Japan Union Hospital of Jilin University approved this study. The human cubital-venous-blood was centrifuged at 3000 rpm to obtain the human sera, followed by 1% diluting into ultrapure-water and stored at −80°C in a refrigerator. The sera sample was firstly dialyzed to remove the inherent Desmin, and then 5% diluted by PBS buffer. The Desmin full-length protein was dissolved into PBS/5%serum solution with final concentration of 15 ng/mL. The resulted mixture was named as Solution D. Different amount of solution D was mixed with 1 mL of Solution C, respectively, stirring for 2 hours. Thus, the concentrations of Desmin in the corresponding mixture were 0.714, 1.364, 2.501, and 4.286 ng/mL, respectively. The variation of PL intensities with the Desmin concentration was recorded for linear fitting. The linear relationship would be accepted when *R*-square value >0.9.

For evaluation the detection limit, 1, 2, 5, and 10 Desmin in PBS/5% serum solution were prepared and then reacted with 1 mL of Solution C. The sensitivity of the assay was examined by calculating the *R*-squares of linear curves.

### 2.5 Interferences assay

The immunoglobulin G (IgG, 1 ng/mL), alpha fetoprotein (AFP, 1 ng/mL), carcinoembryoic antigen (CEA, 1 ng/mL), Desmin (1 ng/mL) + IgG (1 ng/mL), Desmin (1 ng/mL) + AFP (1 ng/mL), and Desmin (1 ng/mL) + CEA (1 ng/mL) were applied for specificity evaluation of the assay by reacting with the anti-Desmin conjugated CQDs. The *R*-squares of the linear curves (PL intensity—Desmin concentration) were recorded.

## 3 Results and discussion

**Figure 1:**
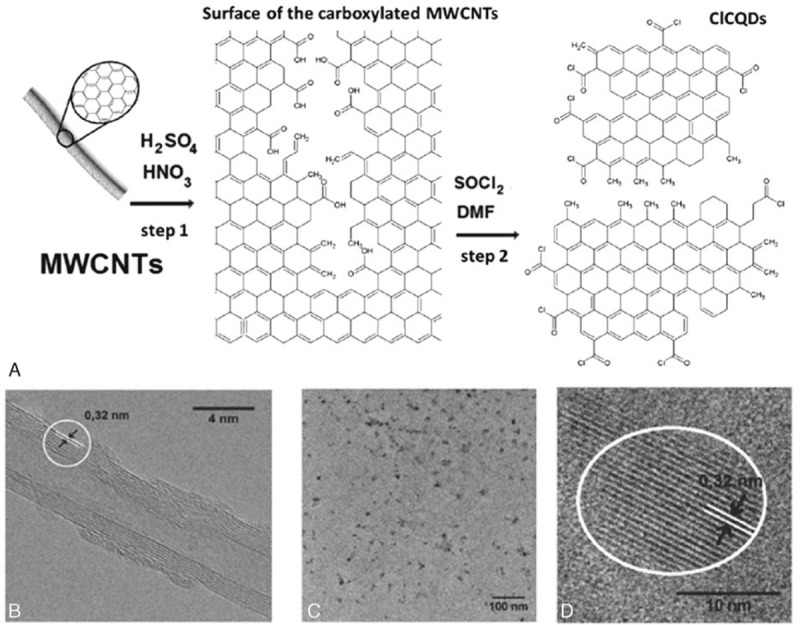
(A) Scheme for the synthesis of ClCQDs; (B) transmission electron microscopy image of MWCNTs; (C) transmission electron microscopy; and (D) high-resolution transmission electron microscopy images of ClCQDs. ClCQDs = carbonyl chloride functionalized carbon quantum dots, DMF = dimethylformamide, MWCNTs = multi-walled carbon nanotubes.

Figure 1A demonstrates the synthesis of ClCQDs. The main approach to functionalizing MWCNTs starts with carboxylation via acid treatment. Heating of MWCNTs in a mixture of concentrated HNO_3_ and H_2_SO_4_ at a 1:3 ratio (v/v) functionalizes the CNTs and partially destroys their sp^2^ structure (Step 1). The MWCNTs could be opened along a line, in a process that resembles the “unzipping” of graphite oxide. This could happen either as a linear longitudinal cut or in a spiraling fashion, depending on the initial site of acid attack and the chiral angle of the CNT. The carboxylic acid functional groups (-COOH) generated on the surface of CNTs constitute the prototype chemical group for surface functionalization. The carbonyl carbon atom covalently binds to the tube and enables exchange of the -OH group with other groups via standard chemical reactions, with consequent attachment of more complex molecules. Reaction with thionyl chloride (SOCl_2_) (Step 2) converts the grafted carboxyl groups, -C(O)OH, to carbonyl chloride groups, -C(O)Cl. The TEM image of the MWCNTs was shown in Figure 1B, and the lattice spacing was about 0.32 nm. Figure 1C displays the TEM images of ClCQDs, which are well dispersed and have similar size. The TEM image in Figure 1D shows that the ClCQDs have average diameter of about 10 nm. The ClCQDs prepared herein have a uniform crystalline structure consisting of parallel crystal planes. This structure resembles the structure of the multiple walls of the MWCNTs before destruction. The image of the CQDs reveals high crystallinity and lattice spacing of also 0.32 nm. Comparing with CQDs synthesized from molecular precursors via bottom-up methods, MWCNTs are also advantageous: they yield highly crystalline nanoparticles bearing sp^2^-structured cores, which should provide better information about how functional groups affect the photoluminescence properties of CQDs.

**Figure 2:**
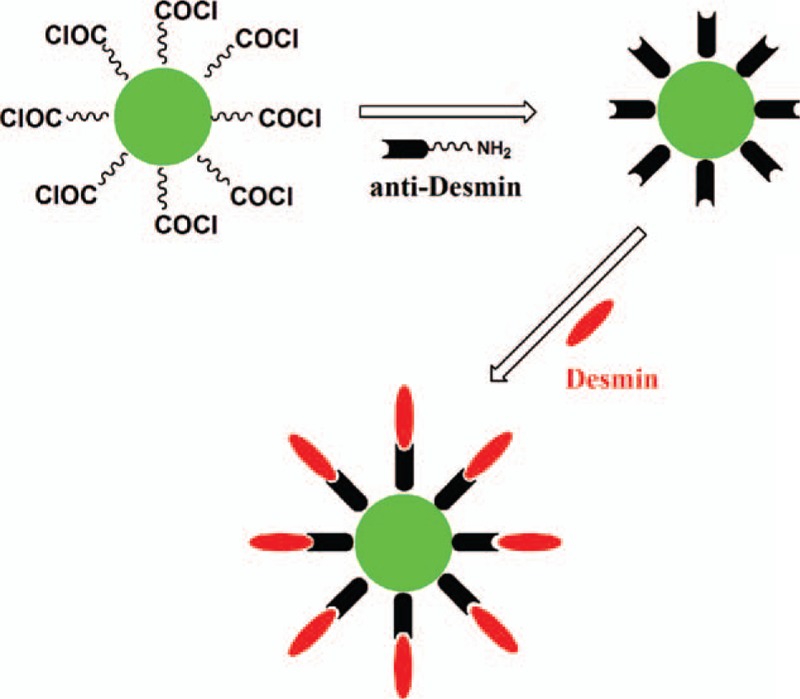
Illustration of the conjugation procedure of anti-Desmin with carbonyl chloride functionalized carbon quantum dot for the detection of Desmin.

The immunofluorescent probe was constructed by conjugation of the ClCQDs and the anti-Desmin. The amino-group of the anti-Desmin protein could be chemically conjugated with the acyl chloride groups on the CQDs, and the anti-Desmin on the CQDs could further chemically adsorb the Desmin. The schematic mechanism is illustrated in Figure 2.

**Figure 3:**
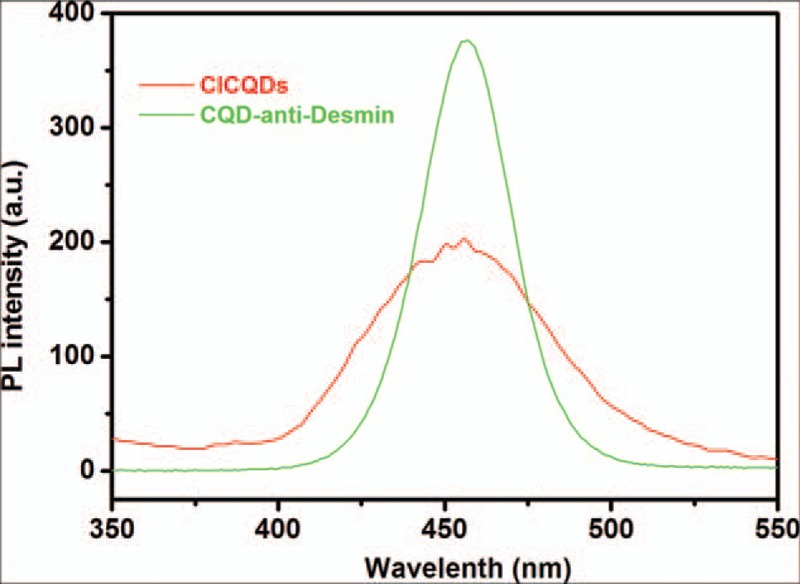
Fluorescence spectra of ClCQDs and CQDs conjugated anti-Desmin. ClCQDs = carbonyl chloride functionalized carbon quantum dots, CQDs =  carbon quantum dots, PL = photoluminescence.

Figure 3 shows the photoluminescence spectra of the prepared ClCQDs, and CQDs conjugated anti-Desmin, which was measured at a fixed excitation wavelength of 300 nm. The emission peak of ClCQDs located at about 452 nm with multicenters, indicating relative complicate distribution. After conjugating with anti-Desmin, the CQDs have much stronger PL intensity at 456 nm than those of ClCQDs. The significant enhancement of photoluminescence could be because of the postpassivated functional amine groups inducing charge delocalization and tuning the carbon work function, which is consist with the previous ref.^[14]^

**Figure 4:**
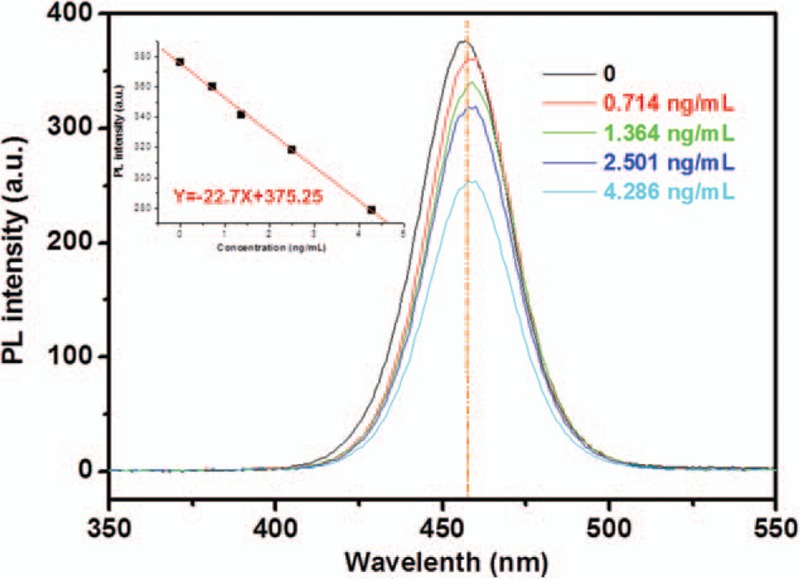
Fluorescence emission spectra of anti-Desmin conjugated carbon quantum dots after reacted Desmin in sera/phosphate buffered saline solution with different concentrations of 0, 0.714, 1.364, 2.501, and 4.286 ng/mL. Inset: the plot of fluorescence intensity against the concentration of Desmin. PL = photoluminescence.

The coverage ratio of the connected Desmin on the probe would affect the fluorescence property by partly quenching the PL intensity. The relationship between the PL intensity of anti-Desmin conjugated CQDs probe and the concentration of Desmin for reaction was established. It can be seen in Figure 4 that the probe without Desmin has the strongest PL intensity of 378.2 at 455 nm. After reaction with the different amount of Desmin, the PL intensity systematically decreased. From the inset of Figure 4, it can be seen that the PL intensity of the probe has good linearity with the Desmin concentration in the solution (from 0.714–4.286 ng/mL), indicating that the Desmin with known concentration could apply for linearly standardizing the PL intensity of a prepared anti-Desmin conjugated CQDs probe in the assay. And thus the Desmin concentrations in unknown serum samples could be determined by the same probe after standardization.

**Figure 5:**
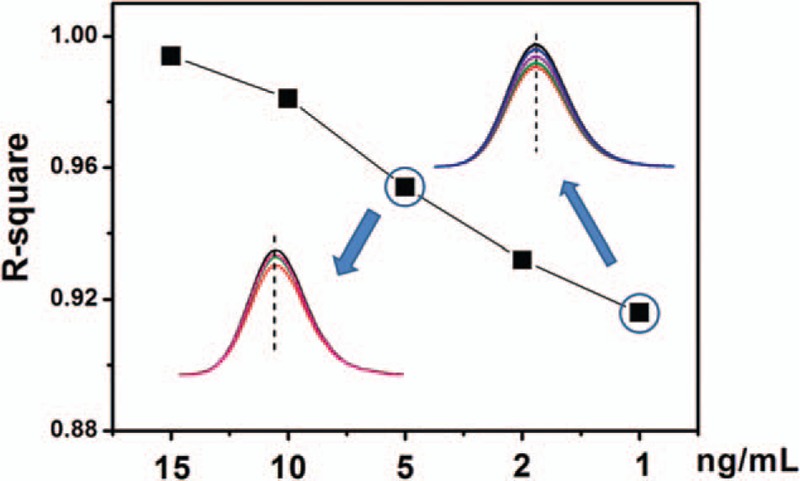
*R*-squares of the fluorescence intensity linear curves of anti-Desmin conjugated carbon quantum dots after reaction with 1, 2, 5, 10, and 15 ng/mL Desmin in 5%serum/phosphate buffered saline solution.

For the early stage screening of CRC, sensitivity of the detection method is of great importance for avoiding diagnosis failure. For evaluation the sensitivity, the prepared anti-Desmin conjugated CQDs probe was also reacted with 1, 2, 5, and 10 ng/mL Desmin in PBS/serum samples, respectively. The calculated *R*-squares of linear curves from relationship between the PL intensities of the reacted probes and Desmin concentrations were shown in Figure 5. The *R*-square value gradually decreased from 0.994 to 0.916 when the Desmin concentration in PBS/serum samples reduced from 15 to 1 ng/mL. Desmin is specifically expressed in mature epithelial cells and secreted into the ideal fluids of lumen at the average concentration of 29.5ng/mL, and the average detective concentration in serum of normal subjects is about 17.5ng/mL. The elevated expression of desmin in CRC tissue and different developmental stages of fetus colon was confirmed by RT-PCR and Western blot analysis from the previous literature,^[8]^ and the reported mean desmin serum levels from patients with CRC, patients with benign bowel disease, and healthy ones had statistically significant difference (105.80 ± 60.30, 71.41 ± 23.47, and 49.21 ± 25.74 ng/mL, respectively). As good linearity of 0.916 for 1 ng/mL Desmin in the assay from Figure 5, a <1 ng/mL Desmin solution could be calibrated and the detection limit of the prepared probe is <1 ng/mL, which completely reaches the testing requirements for Desmin in the human serum, and is suitable for the early evaluation of CRC risk with advantages of cost-effectiveness and sensitivity. Moreover, about 10-fold dilution of serum sample is also available for Desmin detection, which could not only retrench serum sample for clinic medicine application, but also reduce interference of other low expressed proteins, and thus improve the true-positive rate in CRC screening.

**Figure 6:**
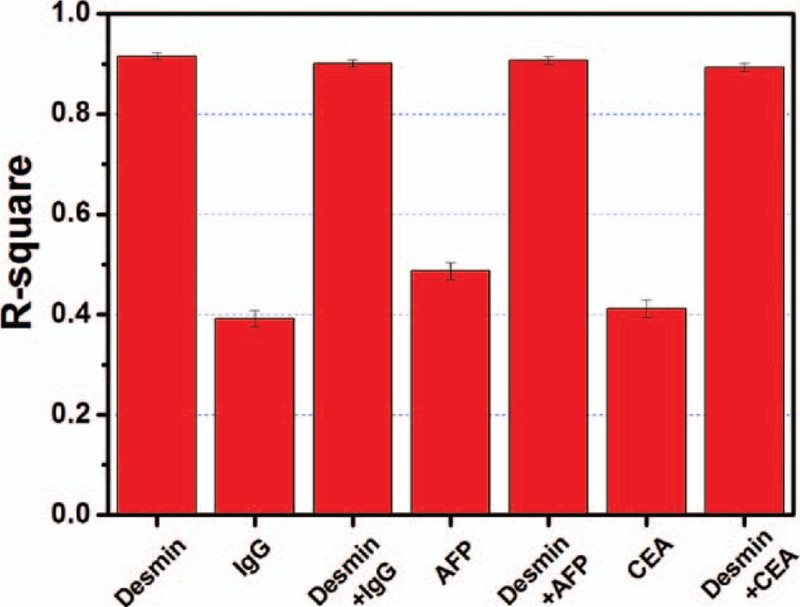
*R*-squares of the fluorescence intensity linear curves of anti-Desmin conjugated carbon quantum dots after reaction with Desmin (1 ng/mL), IgG (1 ng/mL), AFP (1 ng/mL), CEA (1 ng/mL), Desmin (1 ng/mL) + IgG (1 ng/mL), Desmin (1 ng/mL) + AFP (1 ng/mL), and Desmin (1 ng/mL) + CEA (1 ng/mL). AFP = alpha fetoprotein, CEA = carcinoembryoic antigen.

For specificity evaluation, some interferential substances were applied for reacting with the anti-Desmin conjugated CQDs probe, such as IgG, CEA, and AFP. After testing with anti-Desmin conjugated CQDs, the *R*-squares of the fitted lines from PBS/serum solutions with 1 ng/mL Desmin, 1 ng/mL Desmin + 1 ng/mL IgG, 1 ng/mL Desmin + 1 ng/mL AFP, as well as 1 ng/mL Desmin + 1 ng/mL CEA are 0.916, 0.901, 0.907, and 0.893, respectively, as shown in Figure 6, which indicates good linearity. However, the pure 1 ng/mL IgG, 1 ng/mL AFP, and 1 ng/mL CEA after reaction showed no linearity, whose *R*-square values were 0.392, 0.487, and 0.412, respectively. Therefore, it can be deduced that the interferential substances could not connect onto the anti-Desmin conjugated CQDs probe for PL quenching, suggesting excellent specificity of the probe with less susceptibility to the interference protein.

In addition, the synthesis strategy of CQDs probe in this work is very favorable for the mass production, guaranteeing the uniformity of the probe during the picketage and the testing procedure, which is of great significance for satisfying the requirement of CRC screening from large quantities of the specimens and obtaining good result consistency.

## 4 Conclusions

In summary, the -C(O)OH groups modified CQDs were successfully prepared by a facile acid attack on CNTs method. The -C(O)OH groups were further transported into -C(O)Cl groups by SOCl_2_ treating. The obtained ClCQDs were conjugated onto the anti-Desmin and the resulting product inhibits much higher PL intensity, which could be applied in the fast detection of the Desmin in serum with high sensitivity and specificity. This research provides a promising strategy for the evaluation of colorectal cancer risk with low cost and excellent sensing performance.
